# Corneal Cross-Linking in Keratoconus: Comparative Analysis of Standard, Accelerated and Transepithelial Protocols

**DOI:** 10.3390/jcm15020490

**Published:** 2026-01-08

**Authors:** Ruta Jaruseviciene, Ruta Tamuleviciute, Saulius Galgauskas

**Affiliations:** Faculty of Medicine, Vilnius University, Institute of Health Sciences, M. K. Ciurlionio Str. 21, LT 03101 Vilnius, Lithuania

**Keywords:** keratoconus, corneal cross-linking, accelerated CXL, transepithelial CXL, pediatric keratoconus, riboflavin, UVA irradiation

## Abstract

Keratoconus is a progressive, non-inflammatory corneal ectasia characterized by stromal thinning and conical protrusion. Corneal collagen cross-linking (CXL) remains the only proven treatment to halt its progression. This review compares the mechanisms, efficacy, and safety of standard (Dresden), accelerated, and transepithelial (including iontophoretic) protocols, with particular emphasis on pediatric keratoconus. Studies from PubMed, Scopus, and Web of Science were comprehensively reviewed. Standard CXL remains the gold standard due to its strong biomechanical effect and long-term stability. Accelerated protocols reduce treatment time while maintaining comparable outcomes in selected patients, though the stiffening effect may be shallower. Transepithelial and iontophoretic approaches improve comfort and reduce complications but show reduced efficacy. Future perspectives include oxygen supplementation, customized fluence modulation, and pharmacologic enhancers to improve riboflavin diffusion and oxygen availability.

## 1. Introduction

Keratoconus is a bilateral, non-inflammatory and progressive corneal ectasia characterized by spontaneous stromal thinning and asymmetric protrusion of the cornea resembling a cone. It is a degenerative disorder that can cause significant visual impairment, including myopia, irregular astigmatism, and, in advanced stages, even blindness [1]. Despite many studies exploring molecular and genetic mechanisms, the pathophysiology of keratoconus remains poorly understood [2]. Although it was long considered a rare disease, its epidemiology has changed rapidly, in large part due to better diagnostic methods. According to a 2019 meta-analysis that included more than 7 million individuals, the prevalence of keratoconus in the general population is 1.38 per 1000, with men affected slightly more often than women [3]. The disease usually presents in early adolescence and progresses until the third or fourth decade of life, after which it tends to stabilize, although progression may remain variable throughout life [4].

Early in the disease, refractive changes are usually corrected with glasses or contact lenses. At first, soft spherical or toric lenses may be sufficient to correct myopia or regular astigmatism, but as keratoconus progresses, rigid gas-permeable, scleral, or other specialized lenses are often required [5]. In advanced stages, optical correction alone becomes insufficient. In 1997, corneal cross-linking (CXL) was introduced as the first treatment specifically designed to halt the progression of keratoconus [6].

This article presents a comprehensive narrative review of current corneal cross-linking protocols, focusing on their mechanisms, clinical outcomes, and applicability in pediatric keratoconus.

## 2. Materials and Methods

A comprehensive literature search was conducted in PubMed, Scopus, and Web of Science databases up to October 2025. The search strategy used the keywords: “corneal cross-linking” AND “keratoconus” AND (“standard” OR “accelerated” OR “transepithelial” OR “iontophoresis”). Only articles published in English were included. Randomized controlled trials (RCTs), cohort studies, and relevant meta-analyses were prioritized. Reference lists of selected papers were also screened to identify additional studies. In total, 39 publications were included, focusing on the comparison of the standard (Dresden), accelerated, and transepithelial/iontophoretic protocols, with particular attention to pediatric keratoconus management. Generative artificial intelligence tools were used during manuscript preparation. DeepL Pro (Classic Language Model, version 2024.10; DeepL SE, Cologne, Germany) was used for scientific English language refinement. ChatGPT (version 5 Plus; OpenAI, San Francisco, CA, USA) was used to assist in generating conceptual ideas for visual materials. No AI tools were used for data analysis, interpretation, or generation of scientific results. All AI-assisted content was critically reviewed and approved by the authors.

## 3. Results

### 3.1. The Dresden (Standard) Protocol

The conventional protocol, known as the Dresden protocol, was described by Wollensak and colleagues at Dresden Technical University [7]. It involves patient preparation in the operating room (local anesthesia of the eye using a local anesthetic, e.g., 0.4% oxybuprocaine; plus an antiseptic iodine solution to clean the eye and surrounding skin area), removal of 7–9 mm of central epithelium, stromal saturation with 0.1% riboflavin in 20% dextran for 20 min every minute or for 30 min every 5 min, followed by ultraviolet-A (UVA) irradiation at 370 nm, 3 mW/cm^2^ for 30 min, yielding a total dose of 5.4 J/cm^2^. During irradiation, riboflavin supplementation is continued. Postoperatively, patients receive local antibiotics (0.3% tobramycin), lubricating eye drops, oral analgesics as needed, also a bandage contact lens is used until re-epithelialization (approximately 3 days). Once the epithelium has healed, corticosteroids, e.g., local 1 mg/mL dexamethasone solution, should be applied into the operated eye for 3–4 weeks, plus instillation of lubricating eye drops 4 times a day for 1 month [4,7].

The photochemical principle is based on riboflavin photoactivation, which generates reactive oxygen species (ROS) and creates additional covalent bonds between collagen fibrils. This stiffens the stroma, reduces its deformability, and halts ectatic protrusion [1]. Because riboflavin is a large molecule, it does not penetrate an intact epithelium, which is why epithelial debridement (epi-off) is necessary in this protocol. To ensure endothelial safety, corneal thickness must be at least 400 μm [8].

The Dresden protocol has the strongest evidence base and is highly effective in halting progression, but its disadvantages include pain, delayed epithelial healing, risk of haze, and potential for infection [9].

### 3.2. Accelerated Protocols (A-CXL)

To make treatment faster and potentially safer in thin corneas, accelerated protocols were developed. The steps are similar to those of standard CXL, but differ in that a 0.1% riboflavin 5-phosphate solution is instilled without dextran, the riboflavin solution is instilled every 2 min for 10 min on the removed epithelium and UVA is applied to the eye for a shorter period [4,9]. Accelerated protocols typically use dextran-free formulations to avoid intraoperative thinning, because dextran is known to have an osmotic effect, that leads to corneal thinning and thus to potential endothelial damage [4].

A-CXL keeps the total UVA fluence at 5.4 J/cm^2^ but increases irradiance and shortens exposure time. Several regimens are in use, including 9 mW/cm^2^ for 10 min, 18 mW/cm^2^ for 5 min and 30 mW/cm^2^ for 3–4 min [10,11]. These combinations all target the same fluence but differ in how they stress the oxygen-dependent photochemistry: higher irradiance depletes oxygen more quickly, which can curb cross-link formation unless oxygen is replenished efficiently during exposure [8,9].

Even higher intensities up to 45 mW/cm^2^ for 2 min have been explored [7,9,12]. Based on an ex vivo study of porcine corneas it was demonstrated that irradiation levels up to 45 mW/cm^2^ resulted in significantly higher corneal stiffness compared to non-irradiated control corneas. However, irradiation levels of 50 mW/cm^2^ and above at the corresponding exposure times did not result in significantly increased stiffness [13]. This has been attributed to heightened oxygen consumption and subsequent depletion of oxygen species crucial for the crosslinking process in the cornea [9]. For this reason, high-irradiance protocols are often combined with pulsed UVA light delivery, which alternates brief periods of exposure and rest to allow oxygen replenishment in the corneal stroma. Pulsed A-CXL at 30 mW/cm^2^ for 8 min (1 s on–1 s off) has shown deeper demarcation lines compared to continuous A-CXL at the same fluence, although not all studies agree, and results may vary [7,9].

Clinical evidence supports the overall effectiveness of accelerated protocols, particularly in the first postoperative year. Several randomized controlled trials and meta-analyses have shown that uncorrected and corrected distance visual acuity, keratometric flattening, and stabilization rates are similar between conventional and accelerated CXL, especially for 9 mW/cm^2^ and 18 mW/cm^2^ protocols [9,10]. According to a meta-analysis published in 2019, one of the indicators that was also considered is the demarcation line, which is used to assess the depth of the affected cornea. The deeper the demarcation line, the deeper the area affected by UVA light. As the affected area deepens, the possibility of endothelial cell damage increases. Shorter exposure time reduces the duration of UVA light on already thin stroma and may therefore reduce the risk of endothelial damage, while still allowing stabilization of the ectatic process. Therefore, corneas with a thickness of less than 400 μm should undergo the A-CXL procedure to minimize endothelial cell damage, while corneas thicker than 400 μm could undergo both C-CXL and A-CXL procedures with higher doses to improve the effectiveness of treatment [10]. In addition, A-CXL reduces procedure time, which improves patient comfort and possibly reduces risk of infection [9].

### 3.3. Transepithelial and Iontophoretic CXL

Because epithelial removal is the main cause of postoperative pain and complications, alternative protocols have been developed to preserve the epithelium. Transepithelial or epithelium-on CXL, was first described in 2004 [11]. The principle of TE-CXL is to perform cross-linking without epithelial debridement, thereby improving comfort, accelerating healing, and reducing infection risk. However, the intact epithelium forms a barrier that hinders riboflavin diffusion and oxygen availability, resulting in shallower stromal penetration and reduced biomechanical stiffening compared to epi-off [14].

Riboflavin diffusion could be achieved in several ways: by modifying the permeability of the corneal epithelium, adjusting the physical and chemical properties of the riboflavin molecule, forming an epithelial pocket and thus creating a direct pathway for the riboflavin molecule to enter the corneal stroma. One of the techniques used to achieve diffusion is iontophoresis, which involves the use of an electric current to facilitate the penetration of riboflavin molecules. Trans-epithelial iontophoresis (I-CXL) is a non-invasive method that uses local anesthesia based on the generation of repulsive electromotive forces by applying a weak electric current in an iontophoresis chamber, which increases the penetration of riboflavin into the corneal stroma. The I-CXL procedure is based on the use of low molecular weight riboflavin molecules. These molecules have a molecular weight of 376.40 g/mol, are water-soluble and negatively charged, making them suitable for this type of treatment [7,9,11]. Principle of the procedure: a ring is placed on the eye and filled with a hypoosmolar 0.1% riboflavin solution without dextran; electrodes are connected to the ring; an electric current is applied, starting at 0.2 mA and gradually increasing to 1.0 mA, for a duration of 5 min; The cornea is exposed to 370 nm wavelength UVA light at a distance of 5 cm for 9 min, with a radiation intensity of 10 mW/cm^2^. The electrical current promotes the penetration of the hypoosmolar riboflavin molecule into the corneal stroma [4].

Transepithelial CXL can also be performed without the use of electricity. Other substances can be used for this purpose, such as tris(hydroxymethyl)nitromethane, benzalkonium chloride (BAC), ethylenediaminetetraacetic acid (EDTA), hydroxypropyl methylcellulose (HPMC) [14]. These substances help the riboflavin molecule to penetrate the stroma without removing the epithelium. For example, BAC is a preservative that weakens the corneal epithelial connections and thus improves permeability. It is used in various concentrations (from 0.0075 to 0.02%) in many eyes drop solutions [15]. EDTA works on the same principle as BAC [7].

In 2023 article, Yamagata and Ide decided to increase riboflavin penetration by using 1,2-dioleoyl-3-dimethylammonium-propane (DODAP) and isostearic acid (ISA) instead of MedioCROSS TE, a commercially available riboflavin formulation containing benzalkonium chloride [16]. DODAP is a cationic lipid that has been used to prepare lipid nanoparticles for the delivery of short interfering RNA (siRNA) into cells without significant damage to cells. Therefore, the enhancing effect of DODAP was beneficial for riboflavin delivery as less damage was caused to the corneal epithelial cells than with BAC. The increase in penetration was evaluated using an in vitro corneal epithelial cell culture system by measuring the amount of riboflavin transferred using high performance liquid chromatography. Previous studies have reported that corneal epithelial cells require a long recovery time after BAC-induced damage. BAC has been used as a preservative in eye drop preparations, but several studies have reported that it causes damage to corneal epithelial cells both in vitro and in vivo. These studies report that the damage includes cell detachment and cell lysis at a concentration of 0.01% BAC [15,16]. The combined DODAP/ISA formulation enhanced the riboflavin formulation more effectively than DODAP or ISA alone. When a 10-fold higher concentration of riboflavin was used in the DODAP/ISA formulation, the permeability enhancing effect exceeded that of the MedioCROSS TE formulation. To measure the damage levels of corneal epithelial cells TEER (transepithelial/transendothelial electrical resistance) was used. After 24 h of exposure the TEER of the DODAP/ISA formulation was higher than that of MedioCROSS TE, indicating that the DODAP/ISA formulation was less cytotoxic than MedioCROSS TE [16].

According to a 2023 article published by Liu et al., norepinephrine (NE) has been investigated in preclinical models as a novel pharmacological enhancer for transepithelial CXL [17]. This is based on the ability of NE to transiently loosen epithelial tight junctions, thereby improving corneal permeability for riboflavin. In a murine study, subconjunctival injections of NE at low doses (2–5.5 μL of 1 mg/mL solution) were administered prior to riboflavin application. Following NE pretreatment, riboflavin penetration into the corneal stroma was markedly enhanced. Stromal saturation was confirmed both by histological fluorescence and by OCT imaging, which showed more homogeneous riboflavin distribution compared to untreated controls. The improvement was dose-dependent, with 2 μL identified as the lowest safe and effective dose, while higher doses (5.5 μL) produced more pronounced disruption of epithelial junctions and facilitated rapid riboflavin permeation within thirty minutes. Biomechanical testing performed after UVA irradiation further supported the efficacy of NE-assisted CXL. Corneas exposed to 9 mW/cm^2^ for 2.5 min following NE-enhanced riboflavin loading showed significant increases in elastic modulus and resistance to deformation compared to controls. These parameters reflect improved corneal stiffness, a central therapeutic aim of cross-linking. Notably, endothelial morphology and density remained unchanged, indicating that enhanced penetration did not translate into deeper UVA toxicity. The preservation of endothelial safety while achieving greater stromal stiffening highlights the potential of NE as a clinically viable enhancer. Cell culture experiments in human corneal epithelial cells provided mechanistic insight. NE exposure caused dissociation of the tight-junction marker zonula occludens, directly demonstrating barrier loosening at the cellular level. This finding correlated with the in vivo observations of increased riboflavin diffusion and confirmed that the mechanism of action lies in transient disruption of intercellular junctions rather than permanent epithelial damage. Taken together, these results suggest that norepinephrine may overcome one of the main limitations of transepithelial cross-linking: insufficient riboflavin penetration. By temporarily opening epithelial junctions without inducing long-term structural or inflammatory changes, NE enables more uniform stromal saturation, which translates into stronger biomechanical stiffening under UVA irradiation. Although these findings are limited to preclinical models, the consistent improvements in elastic modulus, deformation resistance, and stromal distribution of riboflavin provide a compelling rationale for clinical translation. Future human trials are needed to determine optimal dosing, safety margins, and long-term efficacy, but norepinephrine currently stands out as a promising adjunct to enhance epi-on CXL [17].

In an article published in 2017, Kandzija and Khutoryanskiy investigated whether encapsulating riboflavin-5′-monophosphate (RbP) into liposomal carriers could enhance its penetration through the intact corneal epithelium, thereby eliminating the need for epithelial debridement during CXL [18]. Using ex vivo bovine corneas mounted in Franz diffusion cells and the “whole-eye” permeation model, the authors compared the diffusion and stromal uptake of two forms of riboflavin: the conventional non-ionic riboflavin (Rb) and its ionic, water-soluble phosphate derivative (RbP), which is currently used clinically in CXL. Interestingly, RbP demonstrated significantly higher corneal penetration (17.3 ± 0.8 µg) compared with Rb (10.4 ± 4.2 µg) after three hours of exposure. High-performance liquid chromatography (HPLC) analysis revealed that once inside the corneal tissue, RbP undergoes enzymatic dephosphorylation to Rb, mediated by phosphatases present in the corneal layers. This enzymatic conversion explains the ability of RbP to act as an efficient precursor of active riboflavin in the stroma, even when epithelial penetration is limited. Six different liposomal formulations of riboflavin-5′-monophosphate, by the conventional film method and the propylene glycol-based polyol dilution method, composed of different phospholipids (egg phosphatidylcholine, soybean S75 and S100) were prepared. However, no statistically significant improvement in stromal drug uptake was achieved compared with water-soluble RbP, regardless of formulation type. Entrapment efficiency of RbP ranged from 3.5% to 9.6% in film-method liposomes and up to 41.8% using the polyol dilution method, reflecting the hydrophilic nature of the drug and poor retention within lipid vesicles. The study concluded that while liposomes are biocompatible and stable carriers, their benefit for hydrophilic drugs like RbP is limited because such molecules are located in the aqueous core and rapidly diffuse out, leading to only a slight improvement in stromal uptake. Although the liposomes exhibited good physicochemical stability, the study concluded that conventional liposomal carriers are ineffective for enhancing transepithelial riboflavin delivery. When the drug was encapsulated in liposomes, the penetration of riboflavin-5′-monophosphate into the cornea did not improve in a statistically significant way. These results can be explained by the hydrophilic properties of riboflavin-5′-monophosphate emphasizing the need for alternative permeability enhancers to achieve sufficient stromal saturation for epi-on CXL [18].

### 3.4. Pediatric Keratoconus

Pediatric keratoconus is typically more aggressive than adult disease and carries a higher risk of rapid progression and early visual loss. Children often present earlier, progress faster, have lower contact-lens tolerance, and a substantial subset may require keratoplasty if progression is not halted, therefore, prompt intervention to stop progression is paramount [19]. Recent evidence consistently supports the role of corneal collagen cross-linking (CXL) as the most effective intervention to halt keratoconus progression in children and adolescents. Across studies, treatment success, defined as stabilization or flattening of keratometry with visual acuity improvement, has been reported in 75–90% of pediatric eyes at 1–10-year follow-up. The overall body of evidence emphasizes that, although pediatric corneas respond similarly to adult eyes initially, they show a greater risk of late regression, highlighting the importance of long-term follow-up and potential retreatment in selected cases [9,19,20,21,22].

The traditional Dresden protocol remains the benchmark for pediatric CXL. Large prospective and retrospective series confirm its capacity to stabilize KC and improve best-corrected visual acuity (BCVA). In long-term studies disease stabilization occurred in over 80% of treated eyes with progressive improvement in Kmax and BCVA for up to 10 years [19,20]. Importantly, Prasher et al. reported that in the study by Padmanabhan et al., which followed 194 pediatric eyes for up to 6.7 years, significant visual and topographic improvement was observed. However, after four years, several eyes demonstrated features suggesting partial reversal of the CXL effect, indicating that biomechanical stabilization may be shorter-lived in younger corneas [19]. Polido et al. similarly noted that corneal collagen turnover in children may contribute to new keratoconus instability or the need for retreatment, though most eyes remained stable long term [20]. Post-operative pain, delayed epithelial healing, and haze are the most frequent drawbacks of the conventional protocol [19].

Accelerated cross-linking (A-CXL) delivers the same fluence in shorter irradiation times. Pediatric studies using irradiances between 9 mW/cm^2^ for 10 min and 18 mW/cm^2^ for 5 min achieved visual and topographic stabilization equivalent to the standard protocol at short- and mid-term follow-up [9,19]. According to 2022 article published by Polido et al., multiple pediatric studies found no significant differences between A-CXL and conventional CXL over 2–4 years, while shorter treatment improved comfort and compliance in younger patients [20]. However, several reports noted shallower demarcation lines and slightly less keratometric flattening with high-irradiance protocols, suggesting a somewhat reduced depth of biomechanical stiffening [9,20]. In pediatric keratoconus, which is characterized by rapid and often aggressive progression, a shallower depth of stromal cross-linking may be associated with an increased risk of late disease recurrence, despite the overall favorable safety profile of accelerated protocols. Overall, A-CXL is considered as a safe and effective option in the pediatric population when shorter procedures or enhanced comfort are priorities.

Using transepithelial protocol and maintaining an intact epithelium reduces pain, haze, and infection risk, but may compromise riboflavin and oxygen diffusion. Prospective pediatric comparative study by Henriquez et al. published in 2017 directly evaluated accelerated transepithelial (epi-on) versus standard epi-off CXL in 61 eyes. After 12 months, both techniques achieved similar visual outcomes, while keratoconus progression occurred in 5,6% of epi-on and 12% of epi-off eyes, no severe adverse events were reported [21]. Evidence summarized by Polido et al. and Prasher et al. indicates that transepithelial CXL achieves lower flattening and higher residual progression than epi-off approaches (approximately 60–70% relative efficacy), but with better postoperative tolerance and fewer complications [19,20]. Iontophoretic (I-CXL) protocols improve riboflavin diffusion through mild electrical current but remain biomechanically weaker, reaching stabilization in roughly 50–75% of pediatric cases [20,22]. These methods may therefore be appropriate for very thin corneas or children unable to tolerate epithelial removal.

Across all protocols, serious complications remain rare. Epi-off techniques carry higher risks of postoperative pain, haze, and sterile infiltrates, whereas epi-on and iontophoretic methods are associated with fewer surface complications but occasionally incomplete biomechanical response [19,20,21]. Prior to initiating treatment, it is essential to ensure adequate control of vernal keratoconjunctivitis and other ocular allergy, followed by prolonged postoperative surveillance to enable early detection of potential disease recurrence [19,20].

Overall, standard and accelerated epi-off CXL protocols remain the most validated and durable treatments for halting pediatric keratoconus. Transepithelial and iontophoretic approaches offer safer, less invasive alternatives when epithelial removal is contraindicated, albeit with modestly reduced efficacy. Emerging oxygen-enhanced, pulsed, and customized-fluence strategies hold promise for improving treatment depth and stability while minimizing morbidity in the pediatric population.

Future perspectives include oxygen supplementation, pulsed UVA delivery, customized fluence modulation, and pharmacological enhancers to overcome oxygen and riboflavin diffusion limitations. Artificial-intelligence-based corneal mapping may also support individualized protocol selection.

### 3.5. Alternative Treatment Strategies

#### 3.5.1. Contact Lens-Assisted Corneal Cross-Linking (CA-CXL)

Contact lens-assisted corneal cross-linking (CA-CXL) is a modification of the conventional epi-off CXL technique designed to safely treat patients with thin corneas by placing a riboflavin-soaked, UV-filter-free soft contact lens on the cornea to artificially increase functional pachymetry above 400 µm [23]. The technique is simple, inexpensive and independent of stromal swelling, achieving approximately 100 µm of additional thickness with the soaked lens and allowing safe UVA irradiation in corneas below the standard threshold for endothelial protection [23,24]. Experimental work demonstrates that CA-CXL achieves approximately 70% of the stiffening effect of the standard Dresden protocol while maintaining strong endothelial protection. Clinical studies further show that CA-CXL can effectively halt keratoconus progression with 70–80% stabilization, along with improvements in visual acuity and corneal topography even in corneas thinner than 400 µm [24]. A comparative densitometry study by Gupta et al. showed that CA-CXL induces minimal and transient corneal haze, peaking at three months and returning to baseline by 6–12 months, in contrast to the more pronounced and prolonged haze seen after standard epi-off CXL [25]. Visual outcomes and keratometric flattening at 12 months were similar across CA-CXL, standard CXL and transepithelial CXL groups, indicating comparable efficacy [25]. Overall, CA-CXL represents as a safe, effective, and accessible alternative for progressive keratoconus in thin corneas, offering biomechanical stabilization with low complication risk.

#### 3.5.2. Oxygen-Enhanced Corneal Cross-Linking

Oxygen-supplemented corneal cross-linking has emerged as an important modification to improve the efficacy of standard CXL protocols by addressing the rapid depletion of stromal oxygen that occurs during UVA irradiation, particularly in accelerated and transepithelial procedures, with studies showing that it can be exhausted within the first 15 s at 3 mW/cm^2^ and within 5 s at 30 mW/cm^2^ in accelerated protocols [22,26,27]. This rapid depletion is important because oxygen dependent type II photochemical pathway is responsible the formation of oxygen radicals and generates singlet oxygen, which is the primary mediator of collagen cross-link formation and makes the CXL effect oxygen dependent [22]. Supplemental oxygen has been shown to increase the biomechanical effect of CXL, deepen the stromal demarcation line and enhance early corneal flattening. A 2022 systematic review and meta-analysis by Borchert et al. demonstrated that increasing oxygen availability during mainly epi-on A-CXL resulted in a significant maximum keratometry reduction of 1.2 diopter at 6 months and an improvement in corrected distance visual acuity of 0.08 logMAR with no serious adverse events reported [26]. The same review summarized that oxygen supplied via goggles, eyelid speculum or facemask resulted in significant reductions in corneal curvature and improved vision compared with room air conditions. It also reported that higher dose oxygen exposure resulted in a deeper stromal demarcation line than low oxygen exposure, indicating a stronger treatment effect [26]. In an experimental work by Yang et al. published in 2024 was shown that biocompatible graphitic carbon nitride (g-C_3_N_4_) quantum dots can generate oxygen under UVA irradiation, helping to counter stromal hypoxia during CXL [27]. Collectively, these findings indicate that oxygen availability is a limiting factor in CXL and that increasing oxygen can enhance its biomechanical and clinical outcomes.

#### 3.5.3. Topography-Guided Photorefractive Keratectomy and Corneal Cross-Linking (CXL+)

Topography-guided corneal cross-linking (CXL+) represents a combined therapeutic approach in which topography-guided photorefractive keratectomy (PRK) is performed to regularize the irregular anterior corneal surface, followed immediately or sequentially by corneal cross-linking (CXL) to stabilize the biomechanically weakened cornea in keratoconus [22,28]. The technique was first described in 2011 by Kanellopoulos and Binder, who introduced a two-step method with CXL performed first and PRK one year later in keratoconus patients [28]. Patients usually underwent two-step procedures: step 1 (topography guided PRK)—epithelial debridement, then—topography-guided PRK is performed to reduce irregular astigmatism and perform partial refractive correction, which corrects approximately 70% of both cylinder and sphere measurements (optical zone diameter is approximately 6 mm; photoablation (selective ablation of tissue based on detailed corneal map, maximum ablation depth 50 µm), application of 0.02% MMC (mitomycin-C) for 10 to 30 s to inhibit cell proliferation and reduce the risk of corneal haze formation, copious irrigation of MMC with balanced salt solution); step 2 (standard CXL) [9,28,29,30]. Studies have evaluated simultaneous (so-called Athens protocol) and sequential approaches. De Rosa et al. reported that simultaneous topography-guided PRK (maximum 50 µm ablation depth) followed by standard CXL significantly improved corrected visual acuity and reduced steepest keratometry over 24 months in mild or moderate keratoconus, concluding that the combined procedure is promising and provide surgeons with another tool to improve vision [28]. A comparative prospective study by Niazi et al. assessed topography-guided vs. non-topography-guided PRK performed either simultaneously (in one session) or sequentially (in two sessions) with CXL, demonstrating that all four groups achieved significant improvements in uncorrected distance visual acuity (UDVA), best corrected distance visual acuity (CDVA), refraction, contrast sensitivity and keratometry, with greater keratometry improvement in the topography-guided groups. However, no statistically significant differences were found in most visual outcomes among the four treatment groups [31]. Topography-guided PRK and CXL combinations are also described in broader refractive surgery reviews, which note that topography-guided PRK aims to normalize the corneal surface by selectively ablating tissue based on detailed corneal maps and may reduce irregular astigmatism more effectively than non-topography-guided approaches, although differences in study design and follow-up contribute to varying results and further comparative evidence is needed [30]. In addition, topography-guided PRK followed by CXL has been reported as one of several CXL-plus, offering potential improvements in corneal symmetry and visual acuity relative to CXL alone [9]. Overall, topography-guided CXL is as a method that can improve functional vision and at the same time halt the ectatic progression, while safety is enhanced by limiting the depth of ablation and applying standard or accelerated CXL immediately afterward. However, the authors emphasize that long-term and comparative studies are needed to prove the superiority of this method over non-topography-guided approaches.

#### 3.5.4. Intracorneal Ring Segments (ICRS) Combined with Corneal Cross-Linking (CXL)

Intracorneal ring segment insertion (ICRS) is described as a minimally invasive additive procedure that flattens the central cornea by shortening the arcs between stromal lamellae and can improve visual acuity in keratoconus without affecting the visual axis [32]. Because ICRS implantation alone does not halt ectatic progression, several authors emphasize that combining ICRS with corneal collagen cross-linking (CXL) may enhance outcomes by coupling refractive regularization with biomechanical stabilization [32,33]. CXL increases stromal resistance through the formation of intra- and interfibrillar covalent bonds induced by riboflavin and UVA irradiation, producing a more stable corneal shape and symmetry. Because these procedures are complementary, combined ICRS + CXL has been proposed to yield greater functional benefits than either modality alone [32]. In prospective comparative study by Nasrat et al. was shown that simultaneous (in one session) and sequential (in two sessions) implantation of ICRS followed by CXL (within one month) both lead to significant postoperative reductions in flat corneal curvature (K1), steep corneal curvature (K2) and mean keratometric (km) readings through 6 months, with no statistically significant differences in keratometric outcomes between the two approaches, indicating that both strategies act safely and effectively to improve corneal curvature [32]. Studies also report that CXL is frequently performed in cases demonstrating progression after ICRS implantation, particularly when increases in simulated or maximum keratometry exceed 0.75 diopters (D) in the preceding six months [34]. However, complications remain a consideration. A retrospective analysis of 333 keratoconic eyes implanted with Keraring segments identified segment extrusion in 2.1% of cases and noted that simultaneous CXL performed in the same session was a possible significant risk factor for extrusion [35]. In a case report published by Nuzzi et al., 31-year-old patient underwent bilateral ICRS implantation combined with CXL due to keratoconus. After nine months, the patient developed extrusion of the ring in the left eye accompanied by corneal thinning and a microperforation into the aqueous chamber, with a remaining irregular astigmatism of 4.50 diopter cyl. 10° [36]. For advanced or asymmetric disease, emerging allogenic alternatives such as corneal allogenic intrastromal ring segments (CAIRS) may also be combined with CXL when progression is present, offering refractive and topographic improvement with reduced risk of complications, such as melt, necrosis, migration, intrusion and extrusion compared with synthetic segments [34,37]. Overall, current evidence supports the concept that ICRS combined with CXL provides improved visual function and biomechanically stabilizing effects, but outcome depends on sequence and timing of CXL, ring type (synthetic or allogenic) and individual patient factors.

## 4. Comparative Summary

A comparative analysis of contemporary corneal cross-linking (CXL) protocols highlights notable differences in procedural technique, biomechanical efficacy, patient comfort, and clinical outcomes. The conventional Dresden protocol, which entails epithelial removal (epi-off) followed by UV-A irradiation at 3 mW/cm^2^ for 30 min, consistently demonstrates the greatest biomechanical reinforcement and deepest stromal penetration, establishing it as the gold standard for halting keratoconus progression. Despite its proven efficacy, the necessity of epithelial removal is associated with increased patient discomfort and prolonged visual recovery. Accelerated CXL protocols, employing higher UV-A intensities (typically 9–18 mW/cm^2^) over shorter durations of 5–10 min, achieve comparable surface-level stiffening while slightly reducing stromal depth. These protocols offer the advantage of reduced procedure times but do not substantially improve post-operative comfort compared to the standard approach.

Transepithelial or iontophoretic protocols, designed to preserve the epithelium (epi-on), markedly enhance patient comfort and facilitate faster recovery. However, the intact epithelial acts as a barrier and limits stromal diffusion of both riboflavin and oxygen, thereby reducing the efficiency of the oxygen-dependent cross-linking reaction, resulting in less stromal stiffening and potentially reduced efficacy in arresting keratoconus progression. In pediatric populations, where disease progression is often rapid and aggressive, conventional or accelerated epi-off protocols remain the preferred choice due to their more predictable long-term outcomes, despite the associated discomfort. Emerging strategies, such as customized partial CXL or combination approaches with refractive procedures, show potential for selected cases; nevertheless, their biomechanical impact and clinical effectiveness are highly patient-dependent and currently supported by limited evidence. Overall, the selection of an appropriate CXL protocol requires careful consideration of the trade-offs between maximal corneal strengthening, procedure duration, patient comfort, and individual clinical characteristics. While the standard Dresden protocol provides the highest biomechanical benefit, accelerated protocols offer efficiency, and transepithelial techniques prioritize patient comfort, underscoring the need for personalized treatment planning in keratoconus management.

**Figure 1:**
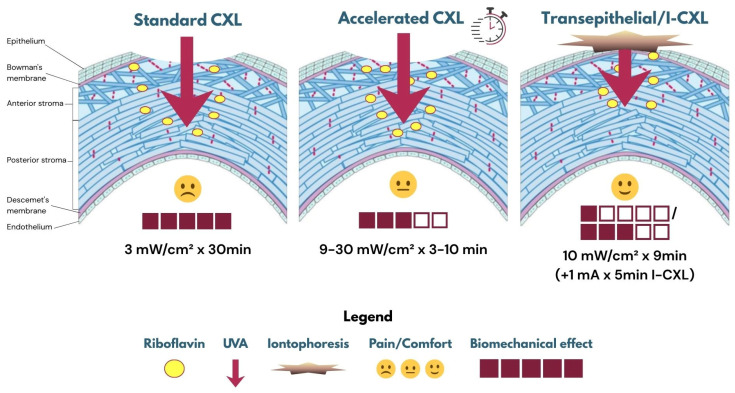
Schematic comparison of standard, accelerated, and transepithelial/iontophoresis-assisted CXL. Relative biomechanical effects (bars) and comfort levels (emoticons) are indicated; riboflavin diffusion and UVA exposure parameters are shown schematically.

A schematic representation of the relative biomechanical effects, riboflavin diffusion, and patient comfort levels for each protocol is shown in Figure 1.

## 5. Discussion and Conclusions

Corneal cross-linking (CXL) has evolved substantially since the introduction of the Dresden protocol. Although the standard epithelium-off method remains the benchmark due to its proven long-term efficacy, it is associated with significant postoperative discomfort, infection risk, and delayed epithelial healing. Accelerated CXL (A-CXL) emerged as a practical modification based on the Bunsen–Roscoe law of reciprocity, aiming to shorten treatment duration without compromising biomechanical outcomes. Nevertheless, clinical results suggest that increased irradiance may reduce oxygen availability within the corneal stroma, potentially limiting cross-link density and long-term stabilization in some cases. Transepithelial and iontophoretic CXL techniques were subsequently developed to improve patient comfort and safety by preserving the epithelial barrier and enhancing riboflavin delivery, yet most studies demonstrate that epithelium-on methods still achieve weaker biomechanical stiffening compared to conventional CXL, particularly in advanced or rapidly progressive keratoconus. The reduced efficacy observed with epithelium-on techniques can therefore be attributed not only to limited riboflavin diffusion but also to decreased oxygen availability within the corneal stroma, which restricts the type II, oxygen-dependent cross-linking pathway. In corneas thinner than 400 μm, transepithelial (epithelium-on) and accelerated corneal cross-linking (A-CXL) protocols are considered safer options, as they reduce epithelial trauma and combine higher irradiance with shorter exposure times and the use of dextran-free riboflavin formulations to minimize intraoperative corneal thinning and endothelial risk. In this context, contact lens-assisted corneal cross-linking (CA-CXL) represents a valuable alternative for the management of progressive keratoconus in thin corneas, enabling safe epi-off cross-linking by temporarily increasing functional pachymetry while providing effective biomechanical stabilization with a low complication profile. Corneal cross-linking remains the cornerstone therapy for halting keratoconus progression, and current evidence indicates that the standard epithelium-off (Dresden) protocol continues to demonstrate the highest biomechanical efficacy and the most reliable long-term stabilization, particularly in children, adolescents, and patients with rapidly progressive disease. In pediatric patients, the disease tends to progress more aggressively, with higher rates of corneal thinning and visual deterioration. Therefore, early intervention is essential. While accelerated and transepithelial techniques offer improved tolerance and safety and provide acceptable results in selected cases, further optimization is required for these approaches to consistently achieve outcomes comparable to conventional CXL. Given the variability in keratoconus presentation, individualized protocol selection based on corneal thickness, patient age, and disease progression rate is essential to achieve optimal visual and biomechanical results. Future research directions include the integration of oxygen supplementation, pulsed or customized UVA fluence delivery, and novel riboflavin formulations designed to enhance stromal diffusion and cross-linking efficiency. In addition, artificial intelligence-assisted corneal mapping and personalized treatment planning may further improve outcomes while minimizing complications. Continued innovation in energy modulation, oxygen dynamics, and cross-linking enhancement strategies is expected to further refine the effectiveness, safety, and accessibility of corneal cross-linking in the coming years.

Compared with transepithelial corneal cross-linking, standard epithelium-off CXL has been shown to induce greater keratometric flattening, reflecting a more pronounced and deeper biomechanical treatment effect. This difference is supported by a meta-analysis of 16 studies published in 2021 by Di et al. including 690 eyes, which demonstrated a significantly larger reduction in maximum keratometry (K_max_) with standard CXL than with transepithelial CXL (weighted mean difference (WMD) −1.12 D; 95% confidence interval (CI) −1.96 to −0.29), while visual acuity, pachymetric measurements, and endothelial parameters were comparable between the two groups at 24 months of follow-up. However, the authors emphasized that larger studies with longer follow-up are required to determine whether these approaches remain comparable in the long term [38]. In contrast, another systematic review published in 2021 by Ng et al., comparing transepithelial and epithelium-off CXL in progressive keratoconus reported no important difference in maximum keratometry at 12 months or longer follow-up, although the certainty of evidence was rated as very low and substantial heterogeneity was present across studies. The analysis found no consistent differences in corrected-distance visual acuity (CDVA) or endothelial cell count between techniques, while epithelium-off CXL was associated with a slightly higher incidence of corneal haze or scarring. Overall, the authors concluded that the available evidence is insufficient to determine whether epi-off CXL provides superior long-term stabilization compared with transepithelial approaches, indicating a need for additional well-designed studies with longer follow-up [39].

## Data Availability

No new data were created or analyzed in this study. Data sharing is not applicable.
